# Syndrome of Undifferentiated Recurrent Fever (SURF): An Emerging Group of Autoinflammatory Recurrent Fevers

**DOI:** 10.3390/jcm10091963

**Published:** 2021-05-03

**Authors:** Riccardo Papa, Federica Penco, Stefano Volpi, Diana Sutera, Roberta Caorsi, Marco Gattorno

**Affiliations:** Center for Autoinflammatory Diseases and Immunodeficiencies, IRCCS Istituto Giannina Gaslini, 16147 Genoa, Italy; riccardopapa@gaslini.org (R.P.); federicapenco@gaslini.org (F.P.); stefanovolpi@gaslini.org (S.V.); dianasutera@icloud.com (D.S.); robertacaorsi@gaslini.org (R.C.)

**Keywords:** autoinflammatory diseases, NGS, SURF, FMF, colchicine, anakinra

## Abstract

Syndrome of undifferentiated recurrent fever (SURF) is a heterogeneous group of autoinflammatory diseases (AID) characterized by self-limiting episodes of systemic inflammation without a confirmed molecular diagnosis, not fulfilling the criteria for periodic fever, aphthous stomatitis, pharyngitis and adenopathy (PFAPA) syndrome. In this review, we focused on the studies enrolling patients suspected of AID and genotyped them with next generation sequencing technologies in order to describe the clinical manifestations and treatment response of published cohorts of patients with SURF. We also propose a preliminary set of indications for the clinical suspicion of SURF that could help in everyday clinical practice.

## 1. Introduction

Syndrome of undifferentiated recurrent fever (SURF) is a heterogeneous group of autoinflammatory diseases (AID) characterized by self-limiting episodes of systemic inflammation without a confirmed molecular diagnosis. First defined by Broderick et al., [1] SURF is increasingly diagnosed in patients with recurrent fever after exclusion of the main hereditary recurrent fevers (HRF) and periodic fever, aphthous stomatitis, pharyngitis and adenopathy (PFAPA) syndrome [2]. Recent evidence suggests the presence of a multi-organ presentation in SURF and, in a relevant percentage of the patients, a complete or at least partial response to colchicine, usually not observed with the same high frequency in PFAPA syndrome [3]. It is possible that omics-based technologies will provide a relevant opportunity to analyse the functional characteristics of immune cells in SURF patients, highlighting the pathological relevance of possible novel genes and supporting the development of new diagnostic tests. On the other hand, the response to colchicine suggests a possible crucial role of cytoskeleton and related proteins, as observed in the other form of HRF responding to this drug, namely the familial Mediterranean fever (FMF) [4]. In this systematic literature review, we will (1) identify a subgroup of patients with SURF among cohorts of patients with suspected AID undergoing next generation sequencing (NGS); (2) describe the clinical manifestations and therapeutic responses of these patients; (3) propose a set of indications for the clinical suspicion of SURF, with the aim of supporting the diagnostic approach in everyday life.

## 2. Materials and Methods

**Figure 1:**
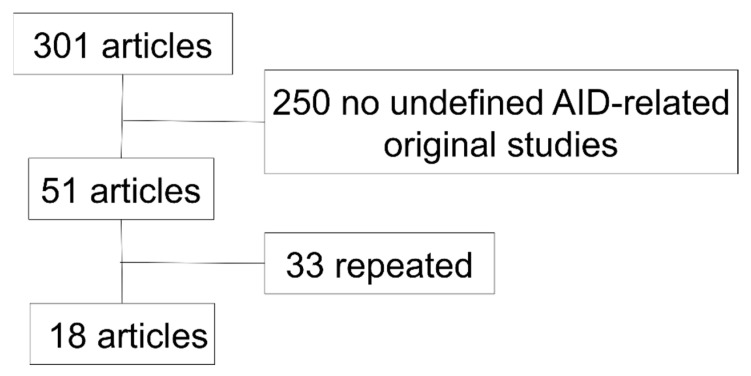
Original English studies found in the PubMed database (https://pubmed.ncbi.nlm.nih.gov; accessed on 2 February 2020) with the queries: “periodic/recurrent fever/s” AND “NGS/Sanger”; “undefined/undifferentiated” AND “autoinflammatory”; “NGS/Sanger” AND “autoinflammatory”. AID, autoinflammatory diseases.

All the original English studies found in the PubMed database (https://pubmed.ncbi.nlm.nih.gov; accessed on 2 February 2020) with the queries: “periodic/recurrent fever/s” AND “NGS/Sanger”; “undefined/undifferentiated” AND “autoinflammatory”; “NGS/Sanger” AND “autoinflammatory”, were included in this review (Figure 1). Excel software was used for the analysis. A descriptive statistical analysis was performed using frequencies and percentages for categorical variables; median and range for numerical variables.

## 3. Results

### 3.1. Studies Selection and Main Characteristics

**Figure 2:**
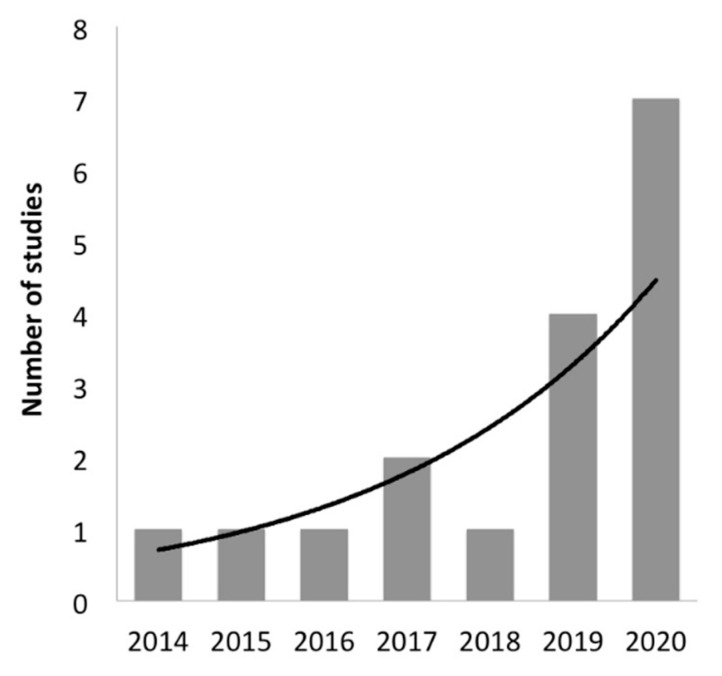
Trend line of studies in Table 1.

**Figure 3:**
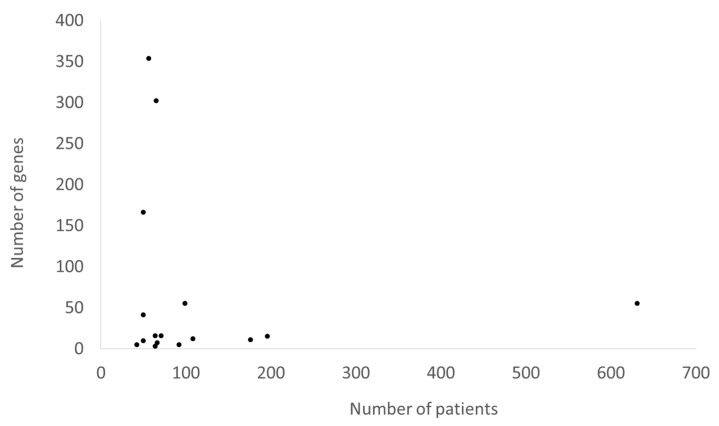
Correlation between the numbers of enrolled patients and analyzed genes of studies in Table 1 except the two using whole exome sequencing.

The main characteristics of the 18 studies regarding the performance of NGS analysis in patients suspected of AID are reported in Table 1. The number of these studies is increased overtime (Figure 2). Recurrent fever has been included in the enrolment criteria by 6/18 (33%) studies. A total of 2179 patients suspected of AID have been genotyped by NGS since 2014. Studies enrolling a large amount of patients usually did not perform an analysis of many genes and vice versa (Figure 3). However, the number of analysed genes in the NGS panels used in the available studies that only referred to AID did not exceed 55. Analysed genes of each study are reported in the Supplementary Table S1. The major enrolled ethnic groups of patients were Caucasian, Middle Eastern and Asian. The exclusion criteria of a previous diagnosis of PFAPA or clinical FMF was informed by the modified Marshall’s criteria and the Tel-Hashomer’s criteria, respectively.

### 3.2. Genotype-Phenotype Assessment

**Figure 4:**
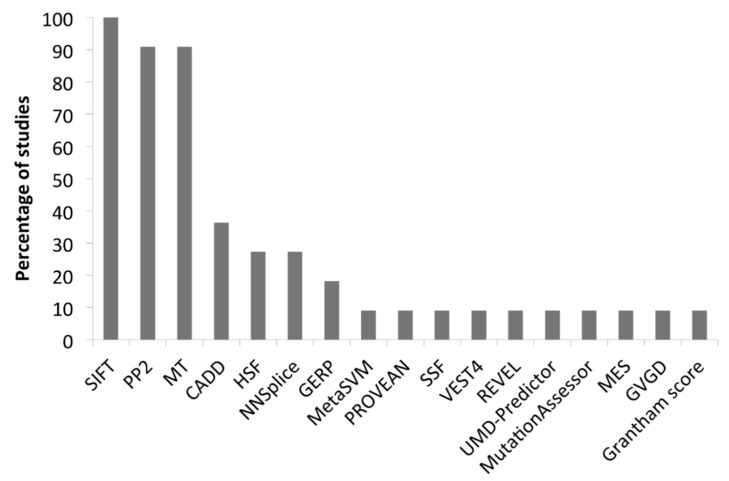
Predictive software of studies in Table 1. SIFT, Sorting Intolerant From Tolerant; PP2, Polymorphism Phenotyping version 2; MT, Mutation Taster; CADD, Combined Annotation Dependent Depletion software; HSF, human splicing finder; NNSplice, Splice Site Prediction by Neural Network; GERP, Genomic Evolutionary Rate Profiling; MetaSVM, Meta-analytic Support Vector Machine; PROVEAN, Protein Variation Effect Analyzer; SSF, Splice Site Finder; REVEL, Rare Exome Variant Ensemble Learner; UMD, Universal Mutation Database; MES, Manufacturing Execution System; GVGD, Grantham Variation and Grantham Deviation.

**Figure 5:**
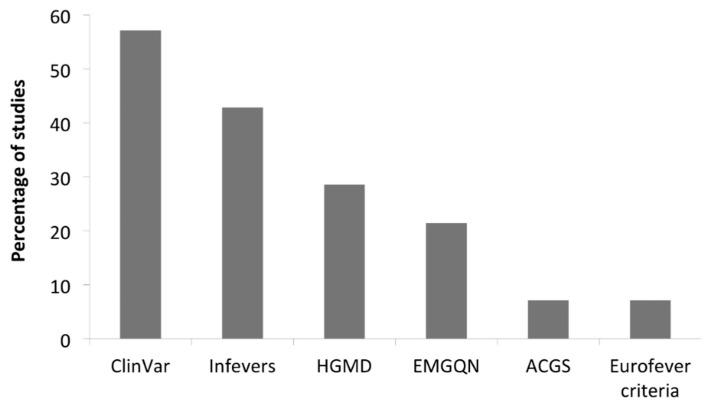
Classification tools of studies in Table 1. HGMD, Human Gene Mutation Database; EMGQN, European Molecular Genetics Quality Network; ACGS, Association for Clinical Genetics Society.

All the analysed studies are reported in Table 1. The assessment of the pathogenicity of each identified variant was obtained by using the minor allele frequency (MAF), predictive software, classification tools and Sanger sequencing confirmation analysis in 12/18 (67%), 11/18 (61%), 14/18 (78%) and 10/18 (56%) studies, respectively. Some studies considered also the pattern of inheritance and available family data. For assessing the MAF, the 1000 Genome Project (http://www.1000genomes.org accessed on 2 February 2021), the Exome Variant Server (http://esv.gs.washington.edu/ESV/ accessed on 2 February 2021), the Exome Aggregation Consortium database (http://exac.broadinstitute.org/ accessed on 2 February 2021) and the Genome Aggregation database (https://gnomad.broadinstitute.org/ accessed on 2 February 2021) were used. Sorting Intolerant from Tolerant (SIFT; https://sift.bii.a-star.edu.sg/ accessed on 2 February 2021) is the most frequently used predictive in silico software (Figure 4), followed by the Polymorphism Phenotyping version 2 (PP2; http://genetics.bwh.harvard.edu/pph2/index.shtml accessed on 2 February 2021) and Mutation Taster (MT; http://www.mutationtaster.org/ accessed on 2 February 2021). Since its first description in 2014, the Combined Annotation Dependent Depletion software (CADD; https://cadd.gs.washington.edu/ accessed on 2 February 2021) is routinely implemented. The most used variant classification tools are ClinVar and the AID-focused website Infevers (https://infevers.umai-montpellier.fr/web/index.php accessed on 2 February 2021) that reports the International Study Group for Systemic Autoinflammatory Diseases (INSAID) variant classification (Figure 5).

### 3.3. Variants Characteristics

In total, more than 1100 variants were reported, ranging from 0.2 to 6.5 per patient. The median rate of detection of a pathogenic or likely pathogenic variant in an undefined AID patient was 20%, ranging from 0% to 89%. Thus, the number of undefined AID patients persists as quite high even if the NGS or the whole exome sequencing (WES) approach has been used (73% in Wang et al.). No studies using a whole genome sequencing approach in undefined AID patients have been published to date.

### 3.4. Clinical Manifestations

As reported in the Methods, patients with suspected AID and undefined recurrent fevers that did not reach a molecular diagnosis after NGS analysis were considered as SURF. Detailed clinical descriptions of 486 SURF patients were available in 5/18 (28%) studies reported in Table 1 and in an additional four specific studies found in the PubMed database.

Clinical features of these patients are reported in Table 2.

The larger cohorts of patients came from the international Eurofever registry, Japan and Middle East [16,19,21]. The median ages at the symptoms onset and patient enrollment are 13 (±13) and 25 (±18) years, respectively. In the four pediatric studies, the median diagnosis delay was 35 months (range 13–78) [5,19,22,23]. Males are 42% of the total. A positive family history ranged from 0% to 32%.

**Figure 6:**
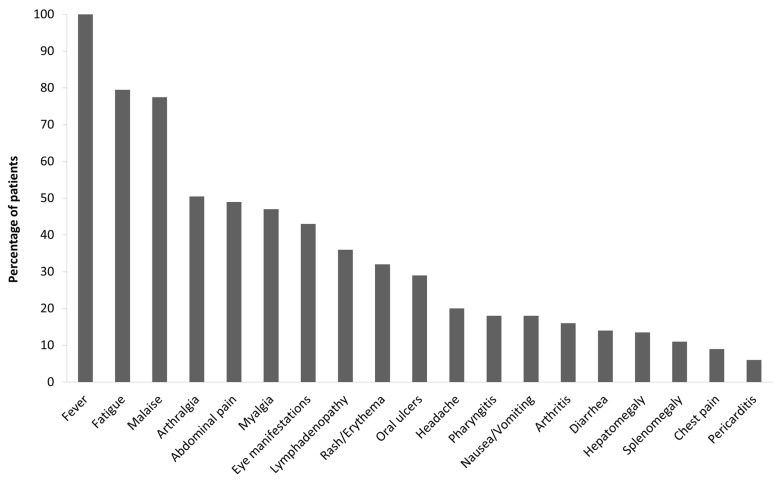
Clinical manifestations of SURF patients reported by at least two studies of Table 2. SURF, syndrome of undifferentiated recurrent fever.

The median duration of inflammatory attacks was 4 ± 1 days with a monthly frequency (11 ± 2 attacks/years). The most frequently reported symptoms during fever attacks were fatigue and malaise (>70% of the patients; Figure 6). Arthralgia, abdominal pain, myalgia and eye manifestations were reported in >40% of the patients. Lymphadenopathy, rash/erythema and oral ulcers were less frequently reported (20–40% of the patients). Headache, pharyngitis, arthritis, nausea/vomiting, diarrhea and hepato/splenomegaly were reported in 10–20% of the patients, and chest pain and pericarditis in less than 10%. Sinusitis, urethritis/cystitis, genital ulcers, gonadal pain, neck stiffness, morning headache, febrile seizure, pleuritis, proteinuria, amyloidosis and sensorineural hearing loss were reported by only single studies.

### 3.5. Treatment Response

**Figure 7:**
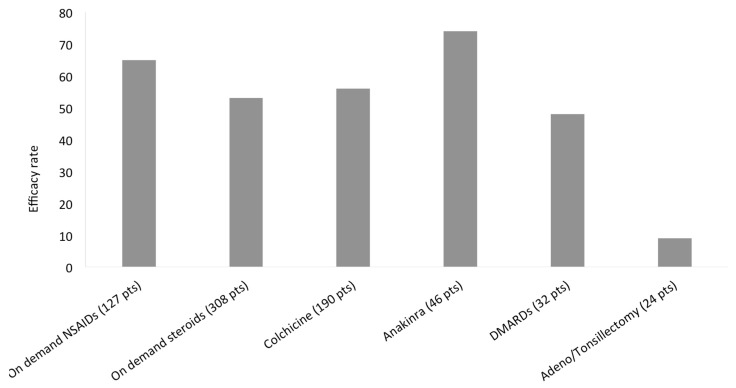
Treatment efficacy in SURF patients. SURF, syndrome of undifferentiated recurrent fever; NSAIDs, non-steroidal anti-inflammatory drugs; DMARDs, disease-modifying anti-rheumatic drug.

The effect of treatment was considered with different methods among the various studies and, herein, any judgement of an evident amelioration of the clinical manifestations after a given treatment. Only a few studies reported a difference between a partial and complete response, and not all authors carefully described the differences between these types of treatment response. Furthermore, on demand or continuous treatment was not always specified. Taking into account these general considerations, the efficacy rate of treatments used in SURF patients is shown in Figure 7. The most frequent treatments were steroids on demand (308 patients) with at least a partial efficacy described in >50% of patients, followed by continuous colchicine treatment (190 patients) and on demand non-steroidal anti-inflammatory drugs (NSAIDs) (127 patients) with a similar efficacy rate (56% and 65%, respectively). Anti-interleukin (IL)-1 treatment (mainly anakinra) was the most effective and frequently used biologic therapy, administered to 46 patients with an efficacy rate of 74%. DMARDs were less frequently used and less effective: 32 patients were treated with different drugs (methotrexate, ciclosporin, azathioprine, mycophenolate mofetil) with an efficacy rate of 48%. Adenoidectomy and tonsillectomy were performed in only 24 patients with a very low efficacy rate (9%).

**Table 2:** Characteristics of SURF patients published in the English literature.

Study	Chandrakasan et al. [5]	Harrison et al. [24]	De Pauli et al. [22]	Ozyilmaz et al. [11]	Ter Haar et al. [21]	Garg et al. [23]	Papa et al. [3]	Hidaka et al. [16]	Demir et al. [19]
Year	2014	2016	2018	2019	2019	2019	2020	2020	2020
Patients	25	11	23	9	180	22	34	133	49
Ethnicity (patients)	Caucasian (14), African (7), others (5)	Caucasian (10), Jewish (1)	Caucasian (20), Middle Eastern (2), others (1)	Middle Eastern	Mixed	Caucasian (11), Asian (5), Jewish (1), African (1), others (4)	Caucasian	Asian	Caucasian, Middle Eastern
Age at enrollment, median (range), years	2.5 (0–9)	ND	4.3 (2–9)	18 (1–47)	ND	ND	ND	39.9 (22–57)	5.9 (3–9)
Age at onset, median (range), years	1.4 (0–5)	35 (24–76)	0 (0–2)	ND	4.3 (1–12) **	0.61 (0–13.5)	ND	33.4 (13–53)	3 (1–6)
Adults onset	0 (0)	11 (100)	0 (0)	0 (0)	65 (35) **	0 (0)	ND	ND	ND
Gender, M:F	16:9	5:6	5:18	5:4	51:49 **	8:14	ND	66:67	34:15
Positive family history	0 (0)	0 (0)	ND	1 (11)	24 (13) **	7 (32)	ND	ND	12 (24)
Attacks/year, median (range)	8 (4–12)	ND	ND	ND	12 (5–14.5)	ND	12 (7–24)	ND ^	10 (6–12)
Attacks duration, median (range), days	4 (3–5)	ND	ND	ND	4 (3–7)	ND	5.9 (4.5–7.3)	ND ^	3 (2–4)
Clinical manifestations	25 (100)	11 (100)	23 (100)	9 (100)	180 (100)	22 (100)	34 (100)	133 (100)	49 (100)
Fever	25 (100)	11 (100)	ND	6 (67)	180 (100)	13 (59)	34 (100)	133 (100)	49 (100)
Abdominal pain	1 (4)	2 (18) ***	12 (52)	8 (89)	87 (48)	4 (18)	17 (50)	ND	31 (63)
Nausea/Vomiting	ND	2 (18) ***	ND	ND	44 (24)	5 (23)	3 (9)	ND	8 (16)
Diarrhea	2 (8)	2 (18) ***	ND	ND	30 (17)	3 (14)	3 (9)	40 (30)	5 (10)
Rash/Erythema	3 (12)	9 (82)	ND	ND	35 (20)	12 (55)	11 (32)	10 (8)	22 (45)
Genital ulcers	ND	1 (9)	ND	ND	ND	ND	ND	ND	ND
Oral ulcers	1 (4)	3 (27)	12 (52)	ND	53 (29)	ND	13 (38)	ND	14 (29)
Pharyngitis/Tonsillitis	1 (4)	ND	13 (57)	ND	47 (18)	ND	13 (38)	ND	5 (10)
Eye manifestations	ND	ND	ND	ND	ND	14 (64)	ND	ND	11 (22)
Arthritis	2 (8)	5 (46)	ND	1 (11)	12 (7)	12 (55)	7 (21)	ND	4 (8)
Arthralgia	ND	8 (72)	ND	ND	107 (59)	10 (46)	12 (35)	57 (43)	27 (55)
Myalgia	ND	8 (72)	15 (65)	ND	80 (44)	13 (59)	9 (27)	25 (19)	23 (47)
Headache	1 (4)	5 (46)	ND	1 (11)	67 (37)	1 (5)	7 (20)	ND	10 (20)
Morning headache	ND	ND	ND	ND	22 (12)	ND	ND	ND	ND
Fatigue	ND	11 (100) ***	ND	ND	106 (59)	ND	ND	ND	ND
Malaise	ND	11 (100) ***	ND	ND	99 (55)	ND	ND	ND	ND
Lymphadenopathy	1 (4)	4 (36)	ND	ND	76 (42)	12 (55)	6 (18)	ND	ND
Splenomegaly	ND	ND	ND	ND	20 (11)	ND	5 (15) ***	ND	1 (2)
Hepatomegaly	ND	ND	ND	ND	21 (12)	ND	5 (15) ***	ND	ND
Chest pain	ND	1 (9)	0 (0)	0 (0)	21 (12)	5 (23)	ND	17 (13)	4 (8)
Pericarditis	ND	2 (18)	ND	ND	10 (6)	ND	ND	ND	1 (2)
Urethritis/cystitis	ND	ND	ND	ND	6 (3)	ND	ND	ND	ND
Gonadal pain	ND	ND	ND	ND	3 (2)	ND	ND	ND	ND
Neck stiffness	1 (4)	ND	ND	ND	ND	ND	ND	ND	ND
Sinusitis	ND	6 (55)	ND	ND	ND	ND	ND	ND	ND
Febrile seizure	ND	ND	ND	ND	ND	ND	ND	ND	4 (8)
Pleuritis	ND	ND	ND	ND	ND	ND	ND	ND	1 (2)
Proteinuria	ND	ND	ND	ND	ND	ND	ND	ND	1 (2)
Amyloidosis	ND	ND	ND	ND	ND	ND	ND	ND	1 (2)
Sensorineural hearing loss	ND	ND	ND	0 (0)	ND	ND	ND	ND	0 (0)
Patients with information about the response to treatment	25 (100)	11 (100)	ND	ND	ND	22 (100)	18 (53)	133 (100)	49 (100)
On demand NSAIDs	ND	ND	ND	ND	80/105 (76%)	3/22 (14%)	ND	ND	ND
On demand steroids	ND	6/10 (60%)	16/21 (76%)	ND	85/104 (82%)	11/22 (50%)	17/18 (94%)	29/133 (22%)	ND
Colchicine	15/25 (60%)	0/3 (0)	6/13 (46%)	ND	29/49 (59%)	ND	14/18 (78%)	44/133 (33%)	31/49 (63%)
DMARDs	ND	0/10 (0)	ND	ND	7/10 (70%)	13/22 (59%)	ND	ND	ND
Anakinra	ND	10/11 (90%)	ND	ND	8/13 (62%)	16/22 (73%)	ND	ND	ND
Tonsillectomy/Adenoidectomy	ND	ND	0/12 (0)	ND	2/12 (17%)	ND	ND	ND	ND

Hispanic, Vietnamese, Asian-Indian, Puerto Rican-Filipino-Mixed European; ** including seven patients with a chronic disease course; ^ 57.1% > 1 episodes/months and 54.9% ≤ 3 days; *** not specify. Results are shown as numbers (%) unless stated otherwise. ND, not declared; NSAIDs, non-steroidal anti-inflammatory drugs; DMARDs, disease-modifying anti-rheumatic drugs.

## 4. Discussion

In the present analysis, we systematically reviewed the papers enrolling patients with suspected AID who were extensively genotyped by NGS technology in order to define the clinical manifestations and response to treatment in patients with recurrence of undefined inflammatory attacks, not fulfilling any PFAPA criteria [25,26] and identified under the new term of SURF.

Inflammation is the first sign of immune system activation against pathogens and damage associated molecular patterns (DAMPS) in living organisms. In the case of the occurrence of inborn errors of immunity, the so-called *horror autoinflammaticus* may develop [27]. In the first conditions reported, the most characteristic clinical feature associated with AID was the recurrence of self-resolving fever attacks, namely HFR. However, a subclinical inflammation in affected patients may be associated with long term or life-threatening complications, such as amyloidosis, with an evident impact on quality of life and life expectation. An early diagnosis and a proper treatment may prevent a severe outcome.

Despite the fact that recurrence was implicit in the definition of the original group of HRF (FMF, MKD, TRAPS), the pathogenic mechanisms correlated with the alternation between flares of inflammation and periods of complete wellbeing still represent a dilemma. The existence is hypothesized of an unbalanced up-regulation of the inflammatory response to common hits, followed by a negative feedback able to down-modulate the primary cause of the immune system hyperactivation. This virtuous cycle prevents an early exitus in people with minor defects in the innate immune system that can cause milder AID phenotypes and allows these mutations to be inherited across future generations. The molecular definition of numerous monogenic AID during the last 20 years dramatically increased our knowledge of the pathways and proteins involved in the innate immune system [28]. However, the large amount of patients displaying undefined recurrent fevers even after NGS suggests a need for further discoveries in the field.

In this review, we define a subset of undefined AID patients with recurrent inflammatory attacks and systemic manifestations not fulfilling the typical features of PFAPA syndrome, that represents an homogeneous subgroup of patients with recurrent fevers characterized by the classical triad of pharyngitis, cervical lymph nodes enlargement and aphthosis [25]. Fever is the physiological reaction to an increased concentration of inflammatory cytokines in the blood during an inflammatory response. This systemic inflammation often requires systemic drugs, such as specific cytokine blockers or other therapies able to prevent the unbalanced inflammatory response.

Among these drugs, colchicine is an ancient and well known agent. Colchicine acts as a cytoskeleton stabilizer with an evident efficacy in some HRF, namely FMF [29]. A similar effect has been shown in the present review in the majority of SURF patients treated with this drug [9]. The clinical definition of SURF as a well-defined and homogeneous clinical entity may be useful to further investigate the molecular basis of the role of the cytoskeleton in the activation and regulation of the inflammatory response. Furthermore, future studies may delineate novel treatments able to control the clinical manifestations of SURF.

This literature review has a number of limitations. First, the variability of the inclusion criteria used in the different analysed studies is associated with a relevant heterogeneity of the studied populations. Notably, in some studies, the exclusion of non-autoinflammatory syndrome was not formally specified. Finally, the not-homogeneous distribution of genes included in the different NGS panels cannot exclude that some patients could harbour mutations of some genes related to AID not covered by the panel used for that study. It is worth noting, however, that in all the analysed studies, the NGS panel included at least the four genes most frequently associated with HRF, namely MEFV, MVK, TNFRSF1A and NLRP3.

In conclusion, we reviewed the literature data regarding an emerging group of patients with recurrent fevers distinct from HRF and PFAPA syndrome, now defined as SURF. According to the analysis of the literature, a set of the clinical variables that could help to distinguish SURF from PFAPA and HRF can be empirically proposed (Table 3). A proper statistical analysis comparing a homogeneous group of SURF patients with patients with HRF and PFAPA will allow the creation of evidence-based classification criteria for SURF, with the final aim of favoring the harmonization of future studies in the fascinating field of AID still without a precise clinical and molecular characterization.

## 5. Footnote

The data in this study are derived from a personal interpretation of published data.

## Figures and Tables

**Table 1:** Studies about the NGS analysis in patients suspected of AID.

N°	Study	Date	Enrollment Criteria	Pts	Ethnicity	Genes	MAF	Predictive in Silico Tools	Variant Classification Tools	Sanger Confirmation	Variants	Variants for Pts, Median (Range)	Pts with Clearly Pathogenic Variants	Pts with Likely Pathogenic Variants	Pts with VUS	Pts with Likely Benign or Benign Variants	Pts without Variants
1	Chandrakasan et al. [5]	2014	Periodic fever	66 *	Caucasian (14), African (7), others (5)°	7	ND	ND	Infevers	Yes	44	0.8 (0–4) *	25 (42)	0 (0)	6 (10)	0 (0)	28 (48)
2	De Pieri et al. [6]	2015	Periodic fever with negative or indefinite genetic analysis; PFAPA syndrome with very early onset and/or poor response to steroids or tonsillectomy	42	Caucasian	5	Any	SIFT, PP2, MT, MutationAssesor, HSF, NNSplice	EMGQN	Yes	38	0.9 (0–4)	0 (0)	0 (0)	24 (57)	5 (12)	13 (31)
3	Rusmini et al. [2]	2016	Systemic AID with at least one mutation in one AID-related gene by Sanger sequencing	50 **	Caucasian	10	<5%	SIFT, PP2	ND	Yes	254	5(ND)	23 (68)	7 (21)	4 (12)	0 (0)	0 (0)
4	Nakayama et al. [7]	2017	Clinical diagnosis of AID	108	Asian	12	<1%	ND	ND	Yes	27	0.25(ND)	ND	ND	ND	ND	ND
5	Omoyinmi et al. [8]	2017	Undiagnosed inflammatory diseases with clinician suspicion of a genetic cause and negative conventional genetic tests	50	Mixed	166	<1% ^	SIFT, PP2, MT	ACGS	Only VUS	325	6.5 (1–16)	6 (12)	11 (22)	31 (62)	0 (0)	2 (4)
6	Kostik et al. [9]	2018	Clinical suspicious of primary immunodeficiency with periodic fever	65	ND	302	<3%	SIFT, PP2, MT, CADD	ClinVar	ND	ND	ND	ND	ND	ND	ND	ND
7	Karacan et al. [10]	2019	Symptoms suggestive of a systemic AID; exclusion of typical FMF	196	Middle Eastern	15	<1%	ND	ClinVar, Infevers, HGMD	ND	ND	ND	14 (10)	27 (14)	97 (50) ^§^	97 (50) ^§^	58 (30)
8	Ozyilmaz et al. [11]	2019	Periodic fever	64	Middle Eastern	3	Any	ND	ClinVar	ND	13	0.2 (0–1)	4 (6)	0 (0)	3 (5)	6 (9)	51 (80)
9	Hua et al. [12]	2019	Chinese adults suspected of systemic AID	92	Asian	5	ND	ND	EMGQN, Infevers	ND	49	0.5 (0–4)	5 (5)	0 (0)	33 (36)	0 (0)	54 (59)
10	Boursier et al. [13]	2019	Suspected monogenic AID (except FMF, DADA2 and MKD after March 2018)	631	ND	55	ND	SIFT, PP2, MT, MES, HSF, NNSplice, SSF,	Infevers	ND	176	0.3 (ND)	44 (7)	50 (8)	63 (10)	0 (0)	474 (75)
11	Papa et al. [3]	2020	Pediatric onset systemic AID; exclusion of PFAPA syndrome and others etiologies; negative or not conclusive Sanger sequencing of suspected genes	50	Caucasian	41	<3%	SIFT, MT, FATHMM, MetaSVM, PROVEAN, CADD	ClinVar	Yes	100	2 (0–6)	3 (8)	3 (8)	25 (50)	10 (20)	9 (18)
12	Suspitsin et al. [14]	2020	Periodic fever	56	ND	354	ND	ND	ClinVar	Yes	ND	ND	9 (16) ^§^	9 (16) ^§^	7 (13)	40 (71) ^§^	40 (71) ^§^
13	Sözeri et al. [15]	2020	Symptoms suggestive of a systemic AID; exclusion of FMF, PFAPA syndrome and other common etiologies; positive Eurofever score for MKD, TRAPS and CAPS	71	Caucasian, Middle Eastern	16	<1%	SIFT, PP2, MT, GERP	EMGQN, ClinVar, HGMD, Eurofever criteria	ND	74	1 (0–3)	35 (49)	0 (0)	36 (51) ^§^	36 (51) ^§^	36 (51) ^§^
14	Hidaka et al. [16]	2020	Unexplained fever	176	Asian	11	<1%	ND	ND	ND	ND	ND	29 (17)	0 (0)	53 (30)	0 (0)	94 (53)
15	Kosukcu et al. [17]	2020	Recurrent fever and high C-reactive protein along with clinical features of inflammation with a possible AID; infections excluded; negative analysis of 14 AID-related genes	11	Middle Eastern	WES	<1%	SIFT, PP2, MT, CADD, REVEL, VEST4	ND	ND	ND	ND	4 (36) ^§^	4 (36) ^§^	7 (64)	0 (0)	0 (0)
16	Wang et al. [18]	2020	Pediatric patients suspected of monogenic AID	288	Asian	3/347/WES	<1%	SIFT, PP2, MT, CADD, UMD-Predictor	ClinVar, Infevers, HGMD	Yes	ND	ND	79 (27)	ND	ND	ND	ND
17	Demir et al. [19]	2020	Symptoms suggestive of a systemic AID; exclusion of FMF, PFAPA syndrome, Blau syndrome, infantile sarcoidosis and other common etiologies; positive Eurofever score for MKD, TRAPS and CAPS	64	Caucasian, Middle Eastern	16	<1%	SIFT, PP2, MT, GERP	ClinVar, HGMD	Yes	ND	ND	15 (23)	21 (33) ^§^	21 (33) ^§^	28 (44) ^§^	28 (44) ^§^
18	Rama et al. [20]	2021	Symptoms of AID (>3 attacks, elevated CRP, age of onset <30 years); exclusion of Armenian, Turkish, Sephardic and Arabic when mentioned and other causes of inflammation	99	ND	55	<1%	SIFT, PP2, MT, MES, HSF, NNSplice, GVGD, Grantham score	Infevers	Yes	ND	ND	10 (10) ^§^	10 (10) ^§^	20 (20)	69 (70) ^§^	69 (70) ^§^

* seven patients were not analyzed; Hispanic, Vietnamese, Asian-Indian, Puerto Rican-Filipino-Mixed European; ** 16 patients were not classified; ^ except for the PRF1 p.A91V, TNFRSF1A p.R92Q, and NLRP3 p.V198M variants; ^§^ classification was not specified. Results are shown as numbers (%) unless stated otherwise. ND, not declared; NGS, next generation sequencing; MAF, minor allele frequency; AID, autoinflammatory diseases; FMF, familial Mediterranean fever; PFAPA, periodic fever, aphthous stomatitis, pharyngitis and adenopathy; MKD, mevalonate kinase deficiency; TRAPS, TNF receptor associated periodic syndrome; CAPS, cryopyrin-associated periodic syndrome; ACGS, Association for Clinical Genetics Society; EMGQN, European Molecular Genetics Quality Network; HGMD, Human Gene Mutation Database; CRP, C-reactive protein; VUS, variant of unknown significance; SIFT, Sorting Intolerant From Tolerant; PP2, Polymorphism Phenotyping version 2; MT, Mutation Taster; HSF, human splicing finder; NNSplice, Splice Site Prediction by Neural Network; CADD, Combined Annotation Dependent Depletion software; GERP, Genomic Evolutionary Rate Profiling; MES, Manufacturing Execution System; SSF, Splice Site Finder; FATHMM, Functional Analysis Through Hidden Markov Models; MetaSVM, Meta-analytic Support Vector Machine; PROVEAN, Protein Variation Effect Analyzer; REVEL, Rare Exome Variant Ensemble Learner; UMD, Universal Mutation Database; GVGD, Grantham Variation and Grantham Deviation.

**Table 3:** Proposed empirical indications for the clinical suspicion of SURF.

Mandatory features
Recurrent fever with elevated inflammatory markers ^1^
Negative criteria for PFAPA ^2^
Negative genotype for HRF ^3^
**Additional supporting features**
Monthly attacks
Attacks duration of 3–5 days
Fatigue/malaise
Arthralgia/myalgia
Abdominal pain
Eye manifestations ^4^
Continuous colchicine/anti-IL1 response ^5^

^1^ at least 3 similar episodes of fever of unknown origin in 6 months; ^2^ according to the modified Marshall’s and/or Eurofever criteria. ^3^ not conclusive NGS and/or Sanger sequencing of at least the most commonly associated genes (MEFV, MVK, TNFRSF1A, NLRP3). ^4^ periorbital edema and/or corneal erythema. ^5^ amelioration of symptoms and/or acute phase reactants. PFAPA, periodic fever, aphthous stomatitis, pharyngitis and adenopathy; HRF, hereditary recurrent fever; IL, interleukin.

## Data Availability

All the data of the present review derive from a personal interpretation of published data.

## Supplementary Materials

The following are available online at https://www.mdpi.com/article/10.3390/jcm10091963/s1, Table S1: Analysed genes in the studies of the Table 1.
