# Supplementary material for: Syndrome of Undifferentiated Recurrent Fever (SURF): An Emerging Group of Autoinflammatory Recurrent Fevers
Source: J Clin Med. 2021 May 3;10(9):1963. doi: 10.3390/jcm10091963 (PMC8124817; doi:10.3390/jcm10091963)
Supplement: Supplementary file 1 [file jcm-10-01963-s001.zip › jcm-1147089-SI.pdf]

Journal of
Clinical Medicine

MDPI

Supplementary Material

Syndrome of Undifferentiated Recurrent Fever (SURF): an Emerging Group of Autoinflammatory Recurrent Fevers

Riccardo Papa, Federica Penco, Stefano Volpi, Diana Sutera, Roberta Caorsi and Marco Gattorno *

Center for Autoinflammatory Diseases and Immunodeficiencies, IRCCS Istituto Giannina Gaslini, Genoa, Italy
*Correspondence: marcogattorno@gaslini.org; Tel.: 0039-01056361

Table S1. Analysed genes in the studies of the Table 1.

N°	Study	Analysed genes*
1	Chandrakasan et al. [5]	MEFV, MVK, TNFRSF1A, NLRP3, LPIN2, PSTPIP1, ELANE
2	De Pieri et al. [6]	MEFV, MVK, TNFRSF1A, NLRP3, NLRP12
3	Rusmini et al. [2]	MEFV, MVK, TNFRSF1A, NLRP3, NLRP12, NOD2, PSMB8, PSTPIP1, IL1RN, LPIN2
4	Nakayama et al. [7]	MEFV, MVK, TNFRSF1A, NLRP3, NLRP12, NOD2, PSMB8, PSTPIP1, IL1RN
5	Omoyinmi et al. [8]	See supplementary tables of the study
6	Kostik et al. [9]	See supplementary tables of the study
7	Karacan et al. [10]	MEFV, MVK, TNFRSF1A, NLRP3, NLRP12, NLRC4, LPIN2, NOD2, PLCG2, PSTPIP1, CECR1, CARD14, IL10RA, TMEM173, SLC29A3
8	Ozyilmaz et al. [11]	MEFV, MVK, TNFRSF1A, NLRP3
9	Hua et al. [12]	WES
10	Boursier et al. [13]	See supplementary tables of the study
11	Papa et al. [3]	See supplementary tables of the study
12	Suspitsin et al. [14]	See supplementary tables of the study
13	Sözeri et al. [15]	MEFV, MVK, TNFRSF1A, NLRP3, NLRP12, TNFRSF11A, LPIN2, NOD2, PSMB8, PSTPIP1, IL1RN, CECR1, ELANE, CARD14, IL10RA, IL10RB
14	Hidaka et al. [16]	MEFV, MVK, TNFRSF1A, NLRP3, NLRP12, NOD2, PSMB8, PSTPIP1, IL1RN, NLRC4, PLCG2
15	Kosukcu et al. [17]	WES
16	Wang et al. [18]	WES
17	Demir et al. [19]	MEFV, MVK, TNFRSF1A, NLRP3, NLRP12, TNFRSF11A, LPIN2, NOD2, PSMB8, PSTPIP1, IL1RN, CECR1, ELANE, CARD14, IL10RA, IL10RB
18	Rama et al. [20]	See supplementary tables of the study

*NGS panels including >30 genes were not reported. WES, whole exome sequencing.
